# Role of sulfonylurea administration in sepsis and septic shock patients: A systematic review

**DOI:** 10.1016/j.clinsp.2023.100197

**Published:** 2023-04-24

**Authors:** Taline Lazzarin, Raquel Simões Ballarin, Filipe Welson Leal Pereira, Paula Schmidt Azevedo, Suzana Erico Tanni, Marcos Ferreira Minicucci

**Affiliations:** Internal Medicine Department, Faculdade de Medicina, Universidade Estadual Paulista (UNESP), Botucatu, SP, Brazil

Sepsis is an organic dysfunction caused by a dysregulated host response to infection. While the septic shock is defined as sepsis with sustained hypotension despite adequate fluid resuscitation, requiring vasoactive drugs to maintain mean arterial pressure greater than 65 mmHg plus a serum lactate level greater than 2 mmoL/L^1^.

Systemic vasodilatation is a critical point in the pathogenesis of septic shock that could be reversed with vasoactive drugs. However, hyporesponsiveness to vasopressor agents is a common feature. A proposed mechanism for this inadequate hemodynamic response to a high dose of vasopressors is the vascular smooth muscle membrane potential alteration. Potassium is a vital ion contributor to this potential, and activation of the ATP-dependent Potassium (K-ATP) channel is well understood to induce vasodilation and inhibit vasoconstriction. Sulfonylurea represents a class of medication that promotes inhibition of the K-ATP channel, which has been shown to increase vasoconstriction in septic shock^2^.

To clarify the effects of sulfonylureas in the treatment of septic shock patients, we performed a Systematic Review (SR) according to the Preferred Reporting Items for Systematic Reviews and Meta-Analyses (PRISMA) guidelines. The protocol was published on PROSPERO (ID: CRD42022369998) based on the patients of interest, the intervention to be studied, the comparison of interventions, and the outcome of interest (PICO) methodology^3^^.^ Regarding the use of sulfonylureas, the PICO framework was as follows: patients, adult sepsis or septic shock patients; intervention, use of sulfonylureas; comparison, comparison between sulfonylureas administration and placebo; and outcome, the mortality rate, hospital length of stay, and timing use of vasoactive agents. The eligibility criteria for the inclusion of studies were observational and randomized controlled trials. We had no restrictions on publication date, language, or full-text availability. Unfortunately, we did not find any study with the inclusion criteria to analyze the effectiveness of using sulfonylurea in septic shock patients. Limited knowledge considering secondary outcomes is described in the studies, which is related to a potential modifiable in the biochemistry pathway of disease. However, it still unknown the effect on critical outcomes for those patients (Fig. 1). We did not perform a metanalysis because none of the trials evaluated the previously proposed outcomes. However, it is essential to highlight our findings.

**Fig. 1:**
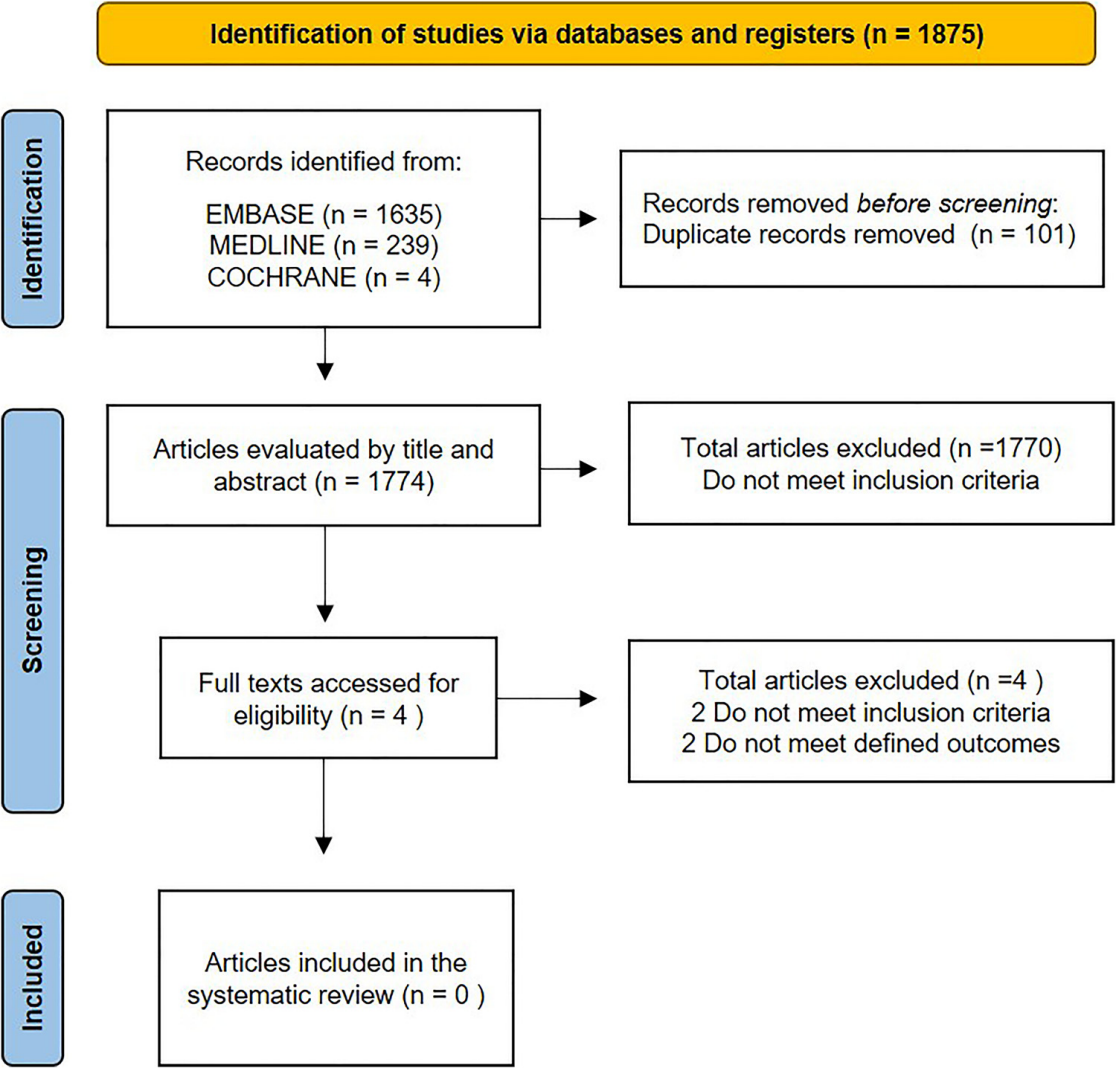
Flowchart for inclusion in the systematic review.

In 2005, Warrillow et al. promoted the first trial to evaluate if sulfonylurea restores norepinephrine responsiveness in patients with septic shock. Patients were randomized to receive glibenclamide (20 mg) or a placebo, and after 24 hours, each patient crossed over to receive the alternative therapy. Glibenclamide failed to achieve a more significant reduction in norepinephrine dose than the placebo. There were also no significant changes in heart rate, mean arterial pressure, and lactate concentration between groups^4^.

In 2007, Morelli et al. performed a clinical study to determine whether different doses of glibenclamide would alter norepinephrine requirements, cardiopulmonary hemodynamics, and global oxygen transport in septic shock. Patients were randomized to receive either 10, 20 or 30 mg of glibenclamide. None of the doses affected cardiopulmonary hemodynamics, global oxygen transport, gas exchange, or electrolytes^5^.

Despite the negative findings of this review, we must not disregard sulfonylureas administration to septic patients considering that no critical outcome such as mortality, length of hospital stays, or time of vasoactive drugs requirement was evaluated. In addition, the two trials were single-center, with small recruited patients and enteral sulfonylurea administration. In experimental studies, the parenteral formulation is the most described, and considering the uncertainty of enteral absorption in shock states, this can be an essential aspect.

In conclusion, more studies should be performed to evaluate better the effects of sulfonylureas administration in patients with sepsis and septic shock.

## Funding

The authors received no financial support for the research, authorship, and/or publication of this article.

## Conflicts of interest

The authors declare no conflicts of interest.
